# Factors Associated With Hospitalization or Intensive Care Admission in Children With COVID-19 in Latin America

**DOI:** 10.3389/fped.2022.868297

**Published:** 2022-04-14

**Authors:** Eduardo López-Medina, German Camacho-Moreno, Martin E. Brizuela, Diana M. Dávalos, Juan Pablo Torres, Rolando Ulloa-Gutierrez, Pio López, Roberto Debbag, Paola Pérez, Jaime Patiño, Ximena Norero, Cristina Mariño, Miguel A. Luengas, Gabriela Ensinck, Carlos Daza, Kathia Luciani, Paola Quintana Kuhner, Mónica Rodriguez, Juan Pablo Rodríguez-Auad, Alejandra Estrada-Villarroel, Mayli Carnevale, Orlando Cesar Mantese, Eitan N. Berezin, José Iván Castillo, Abiel Mascareñas, Andrea Jimenez-Zambrano, Lourdes Dueñas, Mario Melgar, Nancy Galvez, Erika Cantor, Edwin J. Asturias

**Affiliations:** ^1^Centro de Estudios en Infectología Pediátrica, Cali, Colombia; ^2^Department of Pediatrics, Universidad del Valle, Cali, Colombia; ^3^Clínica Imbanaco, Grupo Quirón Salud, Cali, Colombia; ^4^Pediatric Infectious Disease Unit, Fundación HOMI Hospital Pediátrico de la Misericordia and Universidad Nacional de Colombia, Bogotá, Colombia; ^5^Infectious Diseases Division, Hospital Zonal General de Agudos “Dr. Isidoro Iriarte”, Buenos Aires, Argentina; ^6^Faculty of Health Sciences, Universidad Icesi, Cali, Colombia; ^7^Department of Pediatrics, Facultas de Medicina, Hospital Luis Calvo Mackenna, Universidad de Chile, Santiago, Chile; ^8^Servicio de Infectología, Hospital Nacional de Niños “Dr. Carlos Sáenz Herrera”, Centro de Ciencias Médicas de la Caja Costarricense de Seguro Social, San José, Costa Rica; ^9^Department of Infectious Diseases and Epidemiology, Hospital de Pediatría, Buenos Aires, Argentina; ^10^Fundación Valle del Lili, Cali, Colombia; ^11^Hospital del Niño Dr. José Renán Esquivel, Ciudad de Panamá, Panama; ^12^Hospital Militar Central, Bogotá, Colombia; ^13^Servicio de Infectología, Hospital de Niños Víctor J. Vilela de Rosario, Rosario, Argentina; ^14^Hospital Materno Infantil José Domingo de Obaldía, Chiriquí, Panama; ^15^Department of Infectious Diseases, Hospital de Especialidades Pediátricas Omar Torrijos Herrera, Caja de Seguro Social, Ciudad de Panamá, Panama; ^16^Hospital Central del Instituto de Previsión Social, Asunción, Paraguay; ^17^Hospital del Niño “Dr. Ovidio Aliaga Uria”, La Paz, Bolivia; ^18^Hospital Universitario Pediatria Agustin Zubillaga, Barquisimeto, Venezuela; ^19^Hospital de Clínicas da Universidade Federal de Uberlândia, Ubberlandia, Brazil; ^20^Department of Pediatrics, Santa Casa de São Paulo Medical School, São Paulo, Brazil; ^21^Christus Muguerza Hospital Alta Especialidad, Monterrey, Mexico; ^22^Hospital Universitario “Dr. José Eleuterio González”, Universidad Autónoma de Nuevo Leon, Monterrey, Mexico; ^23^Colorado School of Public Health, Center for Global Health, Aurora, CO, United States; ^24^Hospital Nacional de Niños Benjamín Bloom, San Salvador, El Salvador; ^25^Hospital Roosevelt, Ciudad de Guatemala, Guatemala; ^26^Institute of Statistics, Universidad de Valparaíso, Valparaíso, Chile; ^27^Department of Pediatrics, University of Colorado School of Medicine, Aurora, CO, United States

**Keywords:** COVID-19, SARS-CoV-2, children, critical care, hospitalization

## Abstract

**Background:**

Limited data is available from low-middle and upper-middle income countries of the factors associated with hospitalization or admission to pediatric intensive care unit (PICU) for children with COVID-19.

**Objective:**

To describe the factors associated with hospitalization or PICU admission of children with COVID-19 in Latin America.

**Method:**

Multicenter, analytical, retrospective study of children reported from 10 different Latin American countries to the Latin-American Society of Pediatric Infectious Diseases (SLIPE-COVID) research network from June 1, 2020, and February 28, 2021. Outpatient or hospitalized children <18 years of age with COVID-19 confirmed by polymerase chain reaction or antigen detection from the nasopharynx were included. Children with multisystem inflammatory syndrome in children (MIS-C) were excluded. Associations were assessed using univariate and multivariable logistic regression models.

**Results:**

A total of 1063 children with COVID-19 were included; 500 (47%) hospitalized, with 419 (84%) to the pediatric wards and 81 (16%) to the ICU. In multivariable analyses, age <1 year (Odds Ratio [OR] 1.78; 95% CI 1.08–2.94), native race (OR 5.40; 95% CI 2.13–13.69) and having a co-morbid condition (OR 5.3; 95% CI 3.10–9.15), were associated with hospitalization. Children with metabolic or endocrine disorders (OR 4.22; 95% CI 1.76–10.11), immune deficiency (1.91; 95% CI 1.05–3.49), preterm birth (OR 2.52; 95% CI 1.41–4.49), anemia at presentation (OR 2.34; 95% CI 1.28–4.27), radiological peribronchial wall thickening (OR 2.59; 95% CI 1.15–5.84) and hypoxia, altered mental status, seizures, or shock were more likely to require PICU admission. The presence of pharyngitis (OR 0.34; 95% CI 0.25–0.48); myalgia (OR 0.47; 95% CI 0.28–0.79) or diarrhea (OR 0.38; 95% CI 0.21–0.67) were inversely associated with hospital admission.

**Conclusions:**

In this data analysis reported to the SLIPE research network in Latin America, infants, social inequalities, comorbidities, anemia, bronchial wall thickening and specific clinical findings on presentation were associated with higher rates of hospitalization or PICU admission. This evidence provides data for prioritization prevention and treatment strategies for children suffering from COVID-19.

## Introduction

The emergence and rapid global spread of SARS-CoV-2 in early 2020 posed a major threat to populations from low and middle income countries (LMIC), where data on clinical presentations and risk factors for serious COVID-19 infections in children were scarce (1, 2). There are now ~64 million cases of COVID-19 in Latin America (3), with 8–16% of these cases reported among those between 0 and 19 years of age in different countries of the region (4–8). Although COVID-19 presents as mild or asymptomatic in most children, severe disease and hospitalization can occur in ~1% of children and is likely to increase as children represent most of the unvaccinated in these countries (9).

Early identification of factors associated with severe disease in children allows clinicians and public health officials to triage those in need of advanced level of care and is critical as vaccination campaigns are rolled out, allowing the prioritization of those who will benefit the most from early protection. Although several observational studies have provided information on risk factors for hospitalization or critical care admission in children in high-income countries (10–16), detailed demographic, laboratory, radiological and clinical data obtained from a large sample in LMIC countries are scarce.

Pediatric societies around the world have established data registries of pediatric COVID-19 cases to understand the epidemiology, clinical presentation and morbidity and mortality rates in children (9, 17). The Sociedad Latinoamericana de Infectologia Pediatrica (SLIPE) started a multinational collaboration to create a data registry on children being affected with COVID-19 in Latin American countries. The purpose of the present study was to describe the factors associated with hospitalization or pediatric intensive care unit (PICU) admission in children with COVID-19 in Latin America.

## Materials and Methods

Independent pediatricians collected and reported cases of COVID-19 using an online registry created by the SLIPE-COVID network that utilized a standardized data collection form developed in Research Electronic Data Capture (REDCap) hosted by the University of Colorado, Aurora, USA. Investigators from 17 sites in 14 cities from 10 different Latin American countries prospectively identified patients between June 1, 2020, and February 28, 2021 and retrospectively inputted their data. All study sites were level 3 referral hospitals. The registry and study protocol were reviewed and approved by the local institutional review board of each participating center. Since data was collected for routine clinical practice, research was deemed of minimal risk and the requirement for informed consent was waived.

Children <18 years of age who presented to outpatient clinics or hospital emergency departments from participating sites with COVID-19-related symptoms and polymerase chain reaction (PCR) or antigen-confirmed SARS-CoV-2 infection were included. Children with MIS-C were excluded from this analysis.

Case ascertainment varied across sites. Seven cities used an active case surveillance system that allowed to identify children with confirmed COVID-19 from a list of all ambulatory or hospitalized encounters provided by the hospital's epidemiology or microbiology department, while a passive case ascertainment method was used in the remaining seven cities where children were identified after a pediatric infectious disease service consultation was obtained.

Patient characteristics including demographics, clinical, laboratory and radiological data were collected from medical records as documented by the site clinician who evaluated the patient. For children with one or more COVID-19-related consultations during the study period, only data from the first encounter was collected and for children with multiple laboratory reports during their care, only first values were recorded. Nutritional status was classified based on body mass index (BMI) standard deviations (SD) according to the World Health Organization (WHO) into underweight (below −2 SD), normal nutritional status (between −2 and +1 SD), overweight (between +1 and +2 SD) and obesity (above +2 SD) (18). Radiologic findings were categorized according to the density and extent of parenchymal changes. Anemia was defined as hemoglobin below 11 grams/deciliter (19) and thrombocytopenia as platelets < 150,000 × 10^3^ cells/μL (20). C-reactive protein and neutrophil to lymphocyte ratio were dichotomized as previous reports (21–23). For symptoms that were not described in medical records, investigators recorded “non-applicable” when children were too young or too ill to describe their symptoms.

Outcome endpoints for this analysis were divided into (a) outpatient care, (b) hospitalization in a general pediatric ward, or (c) intensive care unit (ICU) admission. Final disposition was at the discretion of primary care team. Data were compared in children requiring admission in the general ward vs. ICU, in those requiring hospitalization (general ward or ICU) vs. outpatient care and in those who did or did not require supplementary oxygen. In the latter two groups laboratory or imaging data were not compared, as children receiving outpatient care seldom had blood drawn or chest X-rays obtained.

### Data Analysis

Summary statistics were used for description of variables. Medians and interquartile ranges (IQR) were used for continuous variables; categorical variables were reported as absolute numbers and proportions. Chi^2^ tests and Fisher's exact tests were used for categorical variables as appropriate, and the Mann-Whitney U test was used for comparing median values of non-normally distributed variables. Associations of baseline characteristics and clinical findings with hospitalization, PICU and/or supplementary oxygen requirement were assessed using univariate and multivariable logistic regression models. Covariates that were significant at the 0.20 level in univariate analysis were included in multivariable models. A backward elimination algorithm was performed to select covariates that were independently associated with hospitalization, PICU and/or supplementary oxygen use, setting a *p* value of <0.05 as significant. Backward variable selection was performed using Rubin's Rules (24). Two multivariate models were created to explore associations with the outcomes of interest. One explored sociodemographic characteristics and comorbidities, and another explored signs/symptoms, laboratory values and radiologic findings. A sensitivity analysis including only children from the seven cities that used an active surveillance system was conducted. In addition, all co-morbidities were integrated in a model that explored the effect of any co-morbidity in the risk of hospital admission or PICU.

For missing data, a multiple imputation chained equation with 90 imputed datasets was used. This value was set using the rule of thumb (25). All variables, including the binary outcomes, were included in the imputation model and further sensitivity analysis was conducted using complete-case data. To control for variability in case ascertainment and non-measured social or cultural confounders, clustered standard errors were estimated to adjust for the correlation between children from the same city in each model. Hospitals from the same city had the same case ascertainment method. Variables that could not be assessed in all children due to age (i.e. anosmia or dysgeusia) were not imputed nor included in multivariable analysis. All statistical analyses were carried out in Stata version 16.0 (StataCorp, College, Station, TX) and *p* values of <0.05 (two-sided) were considered statistically significant. Imputation was not attempted for variables that had more than 50% of their data missing in the complete dataset except for the C-reactive protein values; this variable was imputed given its relevance in predicting severe outcomes in other studies (23, 26).

## Results

### Study Participants

**Figure 1:**
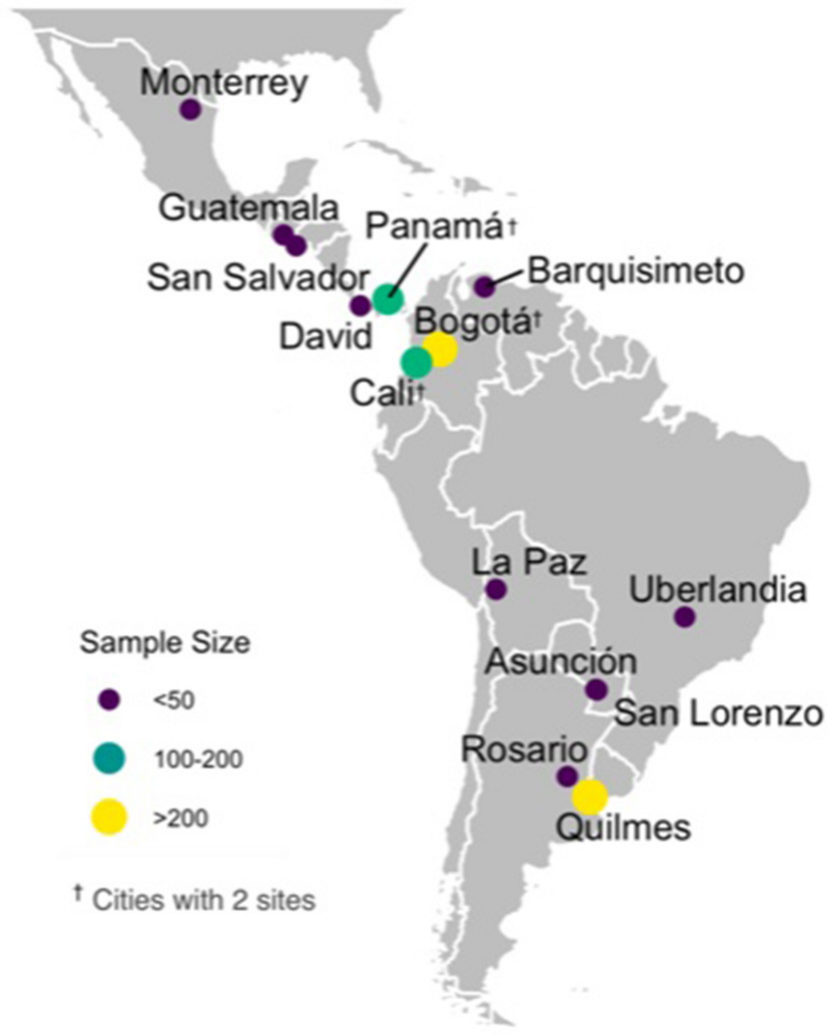
SLIPE-COVID research network-city locations and sites.

A total of 1063 children were included (median age, 3 years [IQR 1–9]; age range 22 days to 17.8 years, 46.4% female, 63% with normal weight and 29% with comorbidity). Of all, 563 (53%) received ambulatory care, while 500 (47%) were hospitalized (419 [84%] in the general ward and 81 [16%] in the ICU) (Table 1). Most children were included from 2 hospitals in Bogotá, Colombia (*n* = 387); 1 hospital in Quilmes, Argentina (*n* = 264); 2 hospitals in Cali, Colombia (*n* = 160) and 2 hospitals in Panamá (*n* = 109). The remaining cases (*n* = 143) were included from Asunción, Paraguay; David, Panamá; Guatemala City, Guatemala; La Paz, Bolivia; Lara, Venezuela; Monterrey, México; Rosario, Argentina; San Lorenzo, Paraguay; San Salvador, El Salvador and Uberlandia, Brazil (Figure 1).

**Table 1:** Demographics and clinical characteristics of pediatric patients with COVID-19.

**Characteristic**	**All patients**	**Outpatient care**	**Hospitalized**	
			**General ward**	**PICU**
	**(*n* = 1,063)**	**(*n* = 563)**	**(*n* = 419)**	**(*n* = 81)**
**Age, median (IQR), yr**	3 (1–9)	4 (1–10)	3 (1–9)	3 (1–9)
**Age groups, yr No. (%)**				
<1	241 (22.7)	109 (19.4)	113 (27.0)	19 (23.5)
1–5	386 (36.3)	214 (38.0)	141 (33.6)	31 (38.3)
6–9	176 (16.6)	95 (16.9)	67 (16.0)	14 (17.3)
≥10	260 (24.5)	145 (25.7)	98 (23.4)	17 (21.0)
**Sex, No. (%)**				
Male	570 (53.6)	291 (51.7)	237 (56.6)	42 (51.9)
Female	493 (46.4)	272 (48.3)	182 (43.4)	39 (48.1)
**Race or ethnic group** ^**a**^ **, No. (%)**				
No.^*^	987	517	396	74
Caucasian	459 (46.5)	285 (55.1)	149 (37.6)	25 (33.8)
Native	16 (1.6)	3 (0.6)	11 (2.8)	2 (2.7)
Black or African American	9 (0.9)	6 (1.2)	3 (0.8)	0 (0.0)
Mixed race	503 (51.0)	223 (43.1)	233 (58.8)	47 (63.5)
**Population, No. (%)**				
No.^*^	880	415	385	80
Urban	807 (91.7)	393 (94.7)	347 (90.1)	67 (83.8)
Rural	73 (8.3)	22 (5.3)	38 (9.9)	13 (16.2)
**Level of Education of caregiver, No. (%)**				
No.^*^	248	115	110	23
No education	87 (35.1)	25 (21.7)	53 (48.2)	9 (39.1)
Primary education	40 (16.1)	17 (14.8)	18 (16.4)	5 (21.7)
High school	79 (31.8)	40 (34.8)	32 (29.1)	7 (30.4)
University degree	42 (16.9)	33 (28.7)	7 (6.4)	2 (8.7)
**Full immunization coverage, No. (%)**				
No.^*^	859	477	310	72
Yes	770 (89.6)	435 (91.2)	274 (88.4)	61 (84.7)
**Nutritional Status** ^**b**^ **, No. (%)**				
No.^*^	562	259	242	61
Underweight	65 (11.6)	17 (6.6)	37 (15.3)	11 (18.0)
Normal Weight	356 (63.3)	173 (66.8)	149 (61.6)	34 (55.7)
Overweight	83 (14.8)	47 (18.1)	31 (12.8)	5 (8.2)
Obese	58 (10.3)	22 (8.4)	25 (10.3)	11 (18)
**Co-morbidities**- **No. (%)**				
Chronic lung disease	123 (11.6)	54 (9.6)	58 (13.8)	11 (13.6)
Congenital heart disease	18 (1.7)	6 (1.1)	9 (2.1)	3 (3.7)
Cirrhosis/Biliary atresia	1 (0.1)	1 (0.2)	0 (0.0)	0 (0.0)
Chronic gastrointestinal disease	9 (0.8)	2 (0.4)	6 (1.4)	1 (1.2)
Renal insufficiency	8 (0.7)	0 (0.0)	8 (1.9)	0 (0.0)
Neurologic disease	59 (5.5)	7 (1.2)	41 (9.8)	11 (13.6)
Metabolic or endocrine disorder	8 (0.7)	1 (0.2)	4 (0.9)	3 (3.7)
Immune deficiency	88 (8.3)	13 (2.3)	57 (13.6)	18 (22.2)
Preterm birth	33 (3.1)	9 (1.6)	17 (4.1)	7 (8.6)
**Any co-morbidity- No. (%)**	305 (28.7)	84 (14.9)	175 (41.8)	46 (56.8)
**Source of infection**- **No. (%)**				
Hospital	27 (2.5)	1 (0.2)	19 (4.5)	7 (8.6)
Community	79 (7.4)	26 (4.6)	42 (10.0)	11 (13.6)
Traveling	5 (0.5)	2 (0.4)	3 (0.7)	0 (0.0)
Home	376 (35.4)	248 (44.0)	113 (27.0)	248 (44.0)
Other	6 (0.6)	4 (0.7)	1 (0.2)	1 (1.2)
Unknown	585 (55.0)	290 (51.5)	247 (58.9)	48 (59.3)
**Symptoms or Signs described or present on admission**- **No. (%)**				
Anosmia No.^*^	25 (3.4) 745, NA = 318	19 (4.7) 403, NA = 160	3 (1.1) 283, NA = 136	3 (5.1) 59, NA = 22
Dysgeusia No.^*^	27 (3.6) 756, NA = 307	19 (4.7) 407, NA = 156	6 (2.1) 289, NA = 130	2 (3.3) 60, NA = 21
Skin rash No.^*^	34 (3.2) 1,059	11 (2.0) 562	21 (5.0) 416	2 (2.5) 81
Conjunctivitis No.^*^	15 (1.4) 1,059	8 (1.4) 562	6 (1.4) 416	1 (1.2) 81
Abdominal pain No.^*^	127 (12.6) 1,011, NA = 51	62 (11.4) 543, NA = 20	54 (13.7) 394, NA = 24	11 (14.9) 74, N = 7
Fever No.^*^	745 (70.1) 1,062	424 (75.3) 563	263 (62.9) 418	58 (71.6) 81
Cough No.^*^	580 (54.7) 1,061	324 (57.5) 563	209 (50.1) 417	47 (58.0) 81
Pharyngitis No.^*^	248 (23.4) 1,059	177 (31.4) 563	59 (14.2) 415	12 (14.8) 81
Rhinitis No.^*^	314 (29.6) 1,060	181 (32.1) 563	111 (26.7) 416	22 (27.2) 81
Headache No.^*^	162 (15.3) 1,058	114 (20.2) 563	39 (9.4) 414	9 (11.1) 81
Myalgia No.^*^	90 (8.5) 1,055	60 (10.7) 561	22 (5.3) 413	8 (9.9) 81
Malaise No.^*^	341 (32.2) 1,058	182 (32.4) 562	123 (29.6) 415	36 (44.4) 81
Diarrhea No.^*^	219 (20.7) 1,059	139 (24.7) 563	61 (14.7) 415	19 (23.5) 81
Vomit No.^*^	199 (18.8) 1,059	107 (19.0) 563	77 (18.6) 415	15 (18.5) 81
Dyspnea No.^*^	231 (21.8) 1,060	41 (7.3) 561	137 (32.8) 418	53 (65.4) 81
Hypoxia No.^*^	175 (16.5) 1,060	8 (1.4) 561	115 (27.5) 418	52 (64.2) 81
Hemoptisis No.^*^	5 (0.5) 1,058	1 (0.2) 561	3 (0.7) 416	1 (1.2) 81
Altered mental status No.^*^	43 (4.1) 1,058	4 (0.7) 561	13 (3.1) 416	26 (32.1) 81
Seizures No.^*^	46 (4.3) 1,058	8 (1.4) 561	21 (5.0) 417	17 (21.2) 80
Dehydration No.^*^	67 (6.3) 1,055	7 (1.2) 560	41 (9.9) 415	19 (23.7) 80
Shock No.^*^	28 (2.6) 1,057	1 (0.2) 559	5 (1.2) 417	22 (27.2) 81
**Onset of symptoms to, median (IQR)**	2 (1–3)	1 (0–3)	1 (0–3)	1 (1–3)

*IQR, Interquartile range; PICU, pediatric intensive care unit*.

a *Race/ethnic group was collected by study personnel based on auto reporting by the study participants. “Mixed race” refers to an individual of mixed European/Native heritage*.

b *Nutritional status was classified based on standard deviations for body mass index according to the World Health Organization into thinness (below −2 standard deviations [SD]), normal nutritional status (between −2 and +1 SD), overweight (between +1 and +2 SD) and obesity (above +2 SD). Body mass index was calculated as weight in kilograms divided by height in meters squared*.

* *Number of patients with available data*.

Most children had fever (70%) and cough (55%). Fewer than one third had signs or symptoms of upper respiratory tract infection (Table 1). Ninety-seven children (9%) had diarrhea without respiratory symptoms while 104 children (9.8%) did not have either fever or respiratory symptoms at admission. The frequency of these clinical presentations varied by age groups (Supplementary Table 1). Frequency of missing data is presented in Supplementary Table 2.

Eighteen children died (median age 6.2 years [IQR 1.8–12.2], 11 [61%] males), 11 (61%) had comorbidities (mainly immune deficiency [*n* = 6, 33%] or neurologic [*n* = 3, 17%]), and 22% came from a rural area. The majority presented with fever (*n* = 15, 83%), hypoxia (*n* = 14, 78%), dyspnea (*n* = 13, 72%) and cough (*n* = 11, 61%). Characteristics of hospital admission among 500 hospitalized children are described in Supplementary Table 3.

### Risk Factors for Hospital Admission

**Figure 2:**
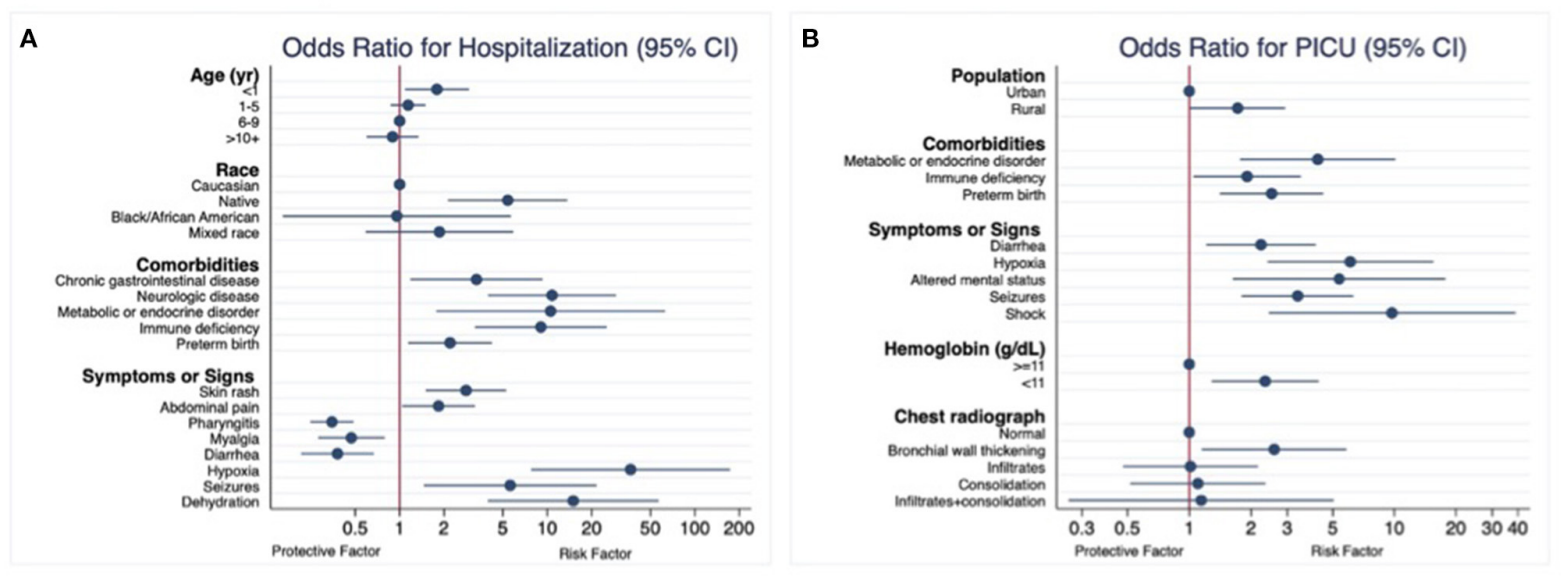
Risk factors among pediatric patients with COVID-19. Multiple imputation chained equations for missing data. **(A)** Hospital admission (general pediatric ward or pediatric intensive care unit). **(B)** Pediatric intensive care admission among hospitalized patients.

Five hundred hospitalized children were compared with 563 children who received outpatient care. In the multivariable model using multiple imputation, younger age (<1 year), native race, and the presence of certain underlying medical conditions were associated with an increased risk for hospital admission (Table 2 and Figure 2). In a model that considered all underlying medical conditions together, the odds for hospital admission were 5.3 times higher (95% CI 3.10 to 9.15) in children with any underlying medical conditions. Both sensitivity analysis (using the complete dataset that included missing data and only children who were enrolled from the seven cities that used an active surveillance system) showed similar results (Supplementary Table 4).

**Table 2:** Demographic and underlying medical conditions associated with hospitalization (general ward and intensive care), among pediatric patients with COVID-19 using multiple imputation chained equations for missing data.

**Characteristic**	**Outpatient care**	**Hospitalization**	**Univariate analysis**	**Multivariable analysis**
	**(*n* = 563)**	**(*n* = 500)**	**OR (95% CI)**	**OR (95% CI)**
**Age groups, yr. No. (%)**				
<1	109 (19.4)	132 (26.4)	1.42 (0.86–2.34)	1.78 (1.08–2.94)
1–5	214 (38.0)	172 (344.4)	0.94 (0.75–1.18)	1.14 (0.87–1.50)
6–9	95 (16.9)	81 (16.2)	Ref.	Ref.
≥10	145 (25.7)	115 (23.0)	0.93 (0.66–1.31)	0.89 (0.60–1.34)
**Sex, No. (%)**				
Male	291 (51.7)	279 (55.8)	Ref.	-
Female	272 (48.3)	221 (44.2)	0.85 (0.66–1.06)	-
**Race or ethnic group** ^**a**^ **, No. (%)**				
Caucasian	285 (55.1)	174 (37.0)	Ref.	Ref.
Native	3 (0.6)	13 (2.8)	6.03 (1.99–18.22)	5.40 (2.13–13.69)
Black or African American	6 (1.2)	3 (0.6)	0.90 (0.11–7.07)	0.96 (0.16–5.66)
Mixed race	223 (43.1)	280 (59.6)	2.02 (0.55–7.14)	1.86 (0.59–5.88)
**Population, No. (%)**				
Urban	393 (94.7)	414 (89.0)	Ref.	-
Rural	22 (5.3)	51 (11.0)	1.95 (1.14–3.35)	-
**Full immunization coverage, No. (%)**				
Yes	435 (91.2)	335 (87.7)	0.63 (0.30–1.35)	-
**Nutritional Status** ^**b**^ **, No. (%)**				
Underweight	17 (6.6)	48 (15.8)	1.57 (0.85–2.90)	-
Normal Weight	173 (66.8)	183 (60.4)	Ref.	-
Overweight	47 (18.1)	36 (11.9)	0.83 (0.54–1.28)	-
Obese	22 (8.5)	36 (11.9)	1.13 (0.67–1.93)	-
**Co-morbidities- No. (%)**				
Chronic lung disease	54 (9.6)	69 (13.8)	1.51 (0.75–3.04)	-
Congenital heart disease	6 (1.1)	12 (2.40)	2.28 (0.55–9.51)	-
Chronic gastrointestinal disease	2 (0.4)	7 (1.40)	3.98 (1.40–11.31)	3.31 (1.18–9.29)
Neurologic disease	7 (1.2)	52 (10.4)	9.22 (3.80–22.34)	10.77 (3.97–29.81)
Metabolic or endocrine disorder	1 (0.2)	7 (1.40)	7.98 (0.99–64.09)	10.53 (1.77–62.79)
Immune deficiency	13 (2.3)	75 (15.0)	7.47 (2.78–20.07)	9.05 (3.24–25.27)
Preterm birth	9 (1.6)	24 (4.8)	3.10 (1.58–6.10)	2.19 (1.14–4.22)

*OR, odds ratio; IQR, interquartile range*.

a *Race/ethnic group was collected by study personnel based on auto reporting by the study participants. “Mixed race” refers to an individual of mixed European/Native heritage*.

b *Nutritional status was classified based on standard deviations for body mass index according to the World Health Organization into thinness (below −2 standard deviations [SD]), normal nutritional status (between −2 and +1 SD), overweight (between +1 and +2 SD) and obesity (above +2 SD). Body mass index was calculated as weight in kilograms divided by height in meters squared*.

Children presenting with skin rash, abdominal pain, dehydration, hypoxia, or seizures were at increased risk for hospital admission, while pharyngitis, myalgia and diarrhea reduced the odds of hospital admission (Table 3). Similar results were obtained from both sensitivity analysis (Supplementary Table 5).

**Table 3:** Signs or symptoms described or present on initial evaluation associated with hospitalization (general ward and intensive care), among pediatric patients with COVID-19 using multiple imputation chained equations for missing data.

**Symptoms or Signs^**a**^ -**	**Outpatient care**	**Hospitalization**	**Univariate analysis**	**Multivariable analysis**
**No. (%)**	**(*n* = 563)**	**(*n* = 500)**	**OR (95% CI)**	**OR (95% CI)**
Anosmia	19 (4.7)	6 (1.8)	-	-
Dysgeusia	19 (4.7)	8 (2.3)	-	-
Skin rash	11 (2.0)	23 (4.6)	2.42 (1.40–4.19)	2.81 (1.51–5.27)
Conjunctivitis	8 (1.4)	7 (1.4)	1.00 (0.47–2.12)	-
Abdominal pain	62 (11.4)	65 (13.9)	1.22 (0.90–1.64)	1.83 (1.04–3.24)
Fever	424 (75.3)	321 (64.3)	0.59 (0.29–1.21)	-
Cough	324 (57.5)	256 (51.4)	0.78 (0.46–1.32)	-
Pharyngitis	177 (31.4)	71 (14.3)	0.36 (0.29–0.45)	0.34 (0.25–0.48)
Rhinitis	181 (32.1)	133 (26.8)	0.77 (0.31–1.90)	-
Headache	114 (20.2)	48 (9.7)	0.42 (0.23–0.77)	-
Myalgia	60 (10.7)	30 (6.1)	0.53 (0.27–1.06)	0.47 (0.28–0.79)
Malaise	182 (32.4)	159 (32.1)	0.98 (0.47–2.08)	-
Diarrhea	139 (24.7)	80 (16.1)	0.58 (0.33–1.03)	0.38 (0.21–0.67)
Vomit	107 (19.0)	92 (18.5)	0.97 (0.55–1.69)	-
Dyspnea	41 (7.3)	190 (38.1)	7.79 (4.61–13.18)	-
Hypoxia	8 (1.4)	167 (33.5)	34.64 (7.58–158.36)	36.69 (7.76–173.39)
Altered mental status	4 (0.7)	39 (7.8)	11.72 (3.57–38.53)	-
Seizures	8 (1.4)	38 (7.6)	5.72 (1.74–18.76)	5.61 (1.46–21.58)
Dehydration	7 (1.2)	60 (12.1)	10.90 (3.85–30.87)	14.99 (3.96–58.84)
Shock	0	27 (5.4)	-	-
**Onset of symptoms to, median (IQR)**	2 (1–3)	1 (0–3)	1.00 (0.96–1.05)	-

a *Laboratory or radiologic data not included due to high frequency of missing data*.

### Risk Factors for ICU Admission

The 81 children who required ICU admission were compared with the remaining 419 children who were hospitalized in the general pediatric ward. In the multiple imputation model, residing in a rural area was the only sociodemographic factor associated with increased odds of ICU care, while metabolic or endocrine disorder, immune deficiency and preterm birth were the underlying medical conditions significantly associated with ICU admission (Table 4). Sensitivity analysis showed similar results (Supplementary Table 6).

**Table 4:** Demographic and underlying medical conditions associated with intensive care, among pediatric patients hospitalized due to COVID-19 using multiple imputation chained equations for missing data.

**Characteristics**	**Place of hospitalization**		**Multiple Imputation**	
	**General ward**	**Pediatric intensive care unit**	**Univariable analysis**	**Multivariable analysis**
	**(*n* = 419)**	**(*n* = 81)**	**OR (95% CI)**	**OR (95% CI)**
**Age groups, yr. No. (%)**				
<1	113 (27.0)	19 (23.5)	0.80 (0.53–1.22)	-
1–5	141 (33.6)	31 (38.3)	1.05 (0.66–1.66)	-
6–9	67 (16.0)	14 (17.3)	Ref.	-
≥10	98 (23.4)	17 (21.0)	0.83 (0.47–1.46)	-
**Sex, No. (%)**				
Male	237 (56.6)	42 (51.9)	Ref.	-
Female	182 (43.4)	39 (48.1)	1.21 (0.73–2.01)	-
**Race or ethnic group** ^**a**^ **, No. (%)**				
Caucasian	149 (37.6)	25 (33.8)	Ref.	-
Native	11 (2.8)	2 (2.7)	1.06 (0.26–4.24)	-
Black or African American	3 (0.8)	0 (0.0)	-	-
Mixed race	233 (58.8)	47 (63.5)	1.22 (0.38–3.88)	-
**Population, No. (%)**				
Urban	347 (90.1)	67 (83.8)	Ref.	Ref.
Rural	38 (9.9)	13 (16.2)	1.76 (1.03–2.99)	1.72 (1.02–2.92)
**Full immunization coverage, No. (%)**				
Yes	274 (88.4)	61 (84.7)	0.70 (0.38–1.28)	-
**Nutritional Status** ^**b**^ **, No. (%)**				
Underweight	37 (15.3)	11 (18.0)	1.28 (0.77–2.14)	-
Normal Weight	149 (61.6)	34 (55.8)	Ref.	-
Overweight	31 (12.8)	5 (8.2)	0.69 (0.25–1.89)	-
Obese	25 (10.33)	11 (18.0)	1.36 (0.60–3.10)	-
**Co-morbidities- No. (%)**				
Chronic lung disease	58 (13.8)	11 (13.6)	0.98 (0.24–3.91)	-
Congenital heart disease	9 (2.1)	3 (3.7)	1.75 (0.54–5.69)	-
Chronic gastrointestinal disease	6 (1.4)	1 (1.2)	0.86 (0.07–10.18)	-
Neurologic disease	41 (9.8)	11 (13.6)	1.45 (0.74–2.83)	-
Metabolic or endocrine disorder	4 (0.9)	3 (3.7)	3.99 (1.35–11.76)	4.22 (1.76–10.11)
Immune deficiency	57 (13.6)	18 (22.2)	1.81 (0.96–3.43)	1.91 (1.05–3.49)
Preterm birth	17 (4.1)	7 (8.6)	2.24 (1.26–3.95)	2.52 (1.41–4.49)

*OR, odds ratio; IQR, interquartile range*.

a *Race/ethnic group was collected by study personnel based on auto reporting by the study participants. “Mixed race” refers to an individual of mixed European/Native heritage*.

b *Nutritional status was classified based on standard deviations for body mass index according to the World Health Organization into thinness (below −2 standard deviations [SD]), normal nutritional status (between −2 and +1 SD), overweight (between +1 and +2 SD) and obesity (above +2 SD). Body mass index was calculated as weight in kilograms divided by height in meters squared*.

Children presenting with diarrhea, hypoxia, altered mental status, seizures or shock were at higher risk for ICU admission. Anemia and bronchial wall thickening in chest radiograph were also associated with increased risk (Table 5). Sensitivity analysis showed similar results (Supplementary Table 7).

**Table 5:** Signs or symptoms, laboratory values and chest radiograph findings described or present on initial evaluation associated with intensive care, among pediatric patients hospitalized due to COVID-19 using multiple imputation chained equations for missing data.

**Symptoms or signs -**	**General ward**	**Pediatric intensive care unit**	**Univariable analysis**	**Multivariable analysis**
**No. (%)**	**(*n* = 419)**	**(*n* = 81)**	**OR (95% CI)**	**OR (95% CI)**
Anosmia	3 (1.1)	3 (5.1)	-	-
Dysgeusia	6 (2.1)	2 (3.3)	-	-
Skin rash	21 (5.0)	2 (2.5)	0.48 (0.15–1.52)	-
Conjunctivitis	6 (1.4)	1 (1.2)	0.84 (0.07–10.47)	-
Abdominal pain	54 (13.7)	11 (14.9)	1.04 (0.36–2.97)	-
Fever	263 (62.9)	58 (71.6)	1.48 (1.06–2.08)	-
Cough	209 (50.1)	47 (58.0)	1.38 (0.74–2.54)	-
Pharyngitis	59 (14.2)	12 (14.8)	1.05 (0.37–3.00)	-
Rhinitis	111 (26.7)	22 (27.2)	1.02 (0.44–2.39)	-
Headache	39 (9.4)	9 (11.1)	1.21 (0.64–2.30)	-
Myalgia	22 (5.3)	8 (9.9)	1.96 (0.52–7.40)	-
Malaise	123 (29.6)	36 (44.4)	1.89 (0.76–4.71)	-
Diarrhea	61 (14.7)	19 (23.5)	1.78 (1.13–2.82)	2.23 (1.21–4.12)
Vomit	77 (18.6)	15 (18.5)	1.00 (0.59–1.71)	-
Dyspnea	137 (32.8)	53 (65.4)	3.89 (1.84–8.25)	-
Hypoxia	115 (27.5)	52 (64.2)	4.73 (2.08–10.80)	6.10 (2.40–15.50)
Altered mental status	13 (3.1)	26 (32.1)	14.50 (5.02–41.92)	5.38 (1.63–17.75)
Seizures	21 (5.0)	17 (21.2)	5.08 (3.30–7.82)	3.37 (1.80–6.32)
Dehydration	41 (9.9)	19 (23.7)	2.90 (1.12–7.50)	-
Shock	5 (1.2)	22 (27.2)	28.69 (11.92–69.04)	9.74 (2.44–38.89)
**Onset of symptoms to, median (IQR)**	1 (0–3)	1 (0–3)	0.99 (0.94–1.04)	-
**Laboratory values**				
Hemoglobin (g/dL)				
No.^*^	372	77		
Median (IQR)	12.3 (10.9–13.5)	11.4 (9.5–13.3)	0.90 (0.81–0.99)	
Anemia^a^ No. (%)	106 (28.5)	34 (44.2)	1.98 (1.25–3.16)	2.34 (1.28–4.27)
Platelets (10^3^ cells/uL)				
No.^*^	391	77	0.998 (0.997–0.999)	
Median (IQR)	299.0 (218–386)	276.0 (157.5–374.5)	2.46 (1.51–4.01)	
Thrombocytopenia^b^ No. (%)	42 (10.7)	18 (23.4)		–
WBC (10^3^ cells/uL)				
No.^*^	397	78		
Median (IQR)	10.0 (6.8–15.0)	12.2 (6.5–14.9)	1.00 (0.97–1.04)	
Neutrophils (10^3^ cells/uL)				
No.^*^	397	76		
Median (IQR)	5.2 (2.5–9.3)	6.0 (3.0–9.3)	0.99 (0.94–1.05)	
Lymphocytes (10^3^ cells/uL)				
No.^*^	394	76		
Median (IQR)	2.8 (1.6–4.6)	3.0 (0.9–5.2)	1.03 (0.96–1.09)	–
Neutrophil to lympho ratio				
No.^*^	393	76		
Median (IQR)	1.8 (0.7–4.3)	1.8 (0.8–4.8)	1.01 (0.95–1.07)	
Values > 5	86 (21.9)	18 (23.7)	1.10 (0.62–1.96)	–
C-reactive protein (mg/dL)				
No.^*^	332	70		
Median (IQR)	12.9 (4.2–39.5)	18.9 (7.0–102.7)	1.004 (1.002–1.006)	
Values > 50 mg/dL. No. (%)	70 (21.1)	23 (32.9)	1.88 (1.16–3.06)	–
**Chest radiograph performed, No. (%)**				
Yes	313 (74.7)	71 (87.65)		
Normal	194 (62.0)	29 (40.8)	Ref.	Ref.
Bronchial wall thickening	28 (8.9)	10 (14.1)	2.27 (0.97–5.28)	2.59 (1.15–5.84)
Interstitial infiltrates	55 (17.6)	16 (22.5)	2.08 (1.19–3.63)	1.01 (0.48–2.16)
Consolidation	23 (7.3)	8 (11.3)	2.53 (1.28–4.98)	1.10 (0.51–2.35)
Interstitial infiltrates + consolidation	13 (4.1)	8 (11.3)	4.32 (1.17–15.93)	1.14 (0.26–5.04)

*WBC, white blood cell count*.

a *Values < 11 g/dL*.

b *Platelets < 150,000 × 10^3^ cells/μ L*.

* *Number of patients with available data*.

### Risk Factors Associated With Supplementary Oxygen Use

Risk factors associated with supplementary oxygen requirement were similar to those associated with hospital admission. In addition, history of chronic lung disease increased the risk for supplementary oxygen requirement (OR 4.45, 95% CI 2.35–8.41) (Supplementary Table 8).

## Discussion

In this multi-country case registry of children with COVID-19 (excluding MIS-C), we identified several factors independently associated with ICU admission, hospitalization and need for supplementary oxygen. Similar to other reports, age under 1-year, comorbidities such as metabolic/endocrine disorders, preterm birth or immune deficiency, certain presenting signs/symptoms and social determinants of health were associated with increased COVID-19-related morbidity (10, 15, 27–30). In addition, low hemoglobin concentrations and bronchial wall thickening on chest radiographs were independently associated with the need for PICU admission.

Social determinants of health have long been recognized to be important predictors as to how epidemics are experienced in terms of infection rates and morbidity (31), particularly in more socially unequal regions, such as Latin America. A multicenter cohort study reported that rates of pediatric PICU admission and deaths due to COVID-19 were higher in Latin American than European children (32). Disparities in health determinants like economic instability, insurance status and housing conditions of patients and their families have consistently placed social, racial, and ethnic minorities at greater risk for severe illness by COVID-19 (31). This is especially true for children and their social determinants influencing life opportunities, disease characteristics and health outcomes. We described greater risks in children with demographic characteristics indicative of lower socio-economic status in Latin America such as native ethnic group or living in rural areas. Social inequalities and low socioeconomic status have also been described as risk factors for death (28), so it is possible that unmeasured socioeconomic or cultural disparities that increase the risk of a more severe or late presentation in children living in rural areas may have been present in this study. For example, although level of education of the caregiver was not imputed or included in multivariable analysis due to the high frequency of missing data, there was a higher proportion of uneducated caregivers among those children who required hospitalization.

Obesity and diabetes mellitus are comorbidities that have been identified as risk factors for disease severity in other studies (12, 15, 33–35). In this study, obesity was not significantly associated with higher risk of hospitalization or ICU admission. However, obesity was more frequent in children requiring ICU admission than in children requiring outpatient care (18 vs. 8.4%, respectively), although no multivariable model comparison was made for these two categories. In addition, obese children were at higher odds of hospitalization (overall and in the ICU), although this was of limited statistical significance. Yet, in studies from high-income countries, obesity has been consistently demonstrated as a risk factor for disease severity in children, especially in adolescents (15, 36). In animal studies, angiotensin-converting enzyme 2 (ACE2) protects against SARS-CoV-2 associated acute respiratory distress syndrome (ARDS) (37). ACE2 expression is decreased in children with diabetes mellitus likely due to glycosylation (38), which may explain their higher risk for hospital or ICU admission in this and other series (33). Although asthma has been suggested as a risk factor for severe illness in children with COVID-19, our study, as well as a registry-based (39) and a cohort study (16) did not confirm this association. Overall, the underlying medical conditions associated with hospital or ICU admission in this Latin American pediatric registry are similar to those described for the same outcomes in US children (39).

As with children with COVID-19 from European and North American countries (11, 40), fever and cough were the predominant clinical features at presentation (70 and 55%, respectively) and approximately a third of children presented with gastrointestinal symptoms. Some clinical manifestations are related to disease progression and complications and are predictive of a higher level of care. As reported in adults (41), children presenting with hypoxia, altered mental status, seizures, shock, dyspnea, or dehydration were more likely to require hospital admission or intensive care. Pharyngitis, myalgia, and diarrhea were identified in this series as inversely associated with hospital admission; and in the complete dataset anosmia and dysgeusia were also found to be protective although of limited statistical significance. A UK study also found that children presenting with upper respiratory signs (rhinorrhea) were less likely to require admission to critical care (35). Preferential distribution of ACE-2 receptors in the upper respiratory or intestinal epithelium (42) may explain the lower frequency of hospitalization in children with these clinical features. Between 9 and 10% of children in this series presented without fever or respiratory symptoms, or only developed diarrhea, findings that need to be considered when developing diagnostic algorithms, especially in settings with actively circulating virus.

Several laboratory findings have been associated with the severity of COVID-19 in adult patients (43). However, data on laboratory values as risk factors for the need for ICU admission in children are scarce. Of routinely collected data in children, only the C-reactive protein has been shown to be a predictor in one study (12), but not in others (44). Similar to our findings, leukocyte indices do not appear to be reliable indicators of disease severity in the pediatric population (45). We found that anemia was the only laboratory predictor for the risk of ICU admission. Whereas this finding has not been described in children, it has been documented as a significant risk factor in adults (46) and should be looked at in detail when assessing children with COVID-19. Like adults (47), bronchial wall thickening was associated with increased risk of ICU admission. Other more severe radiologic abnormalities often described in severe cases (48) were not independently associated with risk of ICU admission in this series, likely due to low number of cases.

Several studies have described risk factors for severe COVID-19 in high-income countries, but data is still scarce from LMIC. Our findings are similar to those from the UK cohort of children for admission to critical care (35). In both studies, children who required PICU were more likely to be of younger age, and had associated comorbidities. Studies from high-income countries have shown that adolescents and patients with elevated C-reactive protein have an increased risk for more severe outcomes (12, 16, 40, 49). These associations were not evident in our data, although different exposure categories and outcome measures may explain these discrepancies.

This study has several limitations. First, seven of the 14 reporting cities used a passive case-ascertainment method which probably introduced a selection bias. Thus, our study population does not represent the full spectrum of COVID-19, but rather children with more severe disease. This is unlikely to impact our results or conclusions given that our goal was to identify factors associated with hospital or ICU admission, and the multilevel regression model clustered by region. To further explore for selection bias, a sensitivity analysis including only children from the seven cities that used an active surveillance system was compared with the analysis that included all children. Factors associated with severity were similar in both analyses suggesting that the effect on our data was minimal.

Second, some relevant variables had missing data, which could reduce the statistical power of the study and produce biased estimates. We tried to overcome this limitation by using multiple imputation, allowing appropriate estimation of the underlying distribution of the data. Results from analyses using the complete dataset (including missing data) and the multiple imputation dataset were similar. Third, we excluded children with MIS-C, who represent one of the most severe clinical forms of pediatric post-infectious COVID-19. A separate full analysis of MIS-C children in Latin American children is ongoing by investigators from the REKAMLATINA network in a cohort of children admitted at several hospitals in the region (50). Finally, due to the multisite nature of the study, full standardization of criteria utilized by all managing clinicians for hospitalization or ICU care was not possible. Therefore, children's outcome disposition may differ by hospitals' capacity and census demands, notwithstanding the severity of the COVID-19 presentation. However, the need for ICU admission or oxygen is an objective measure for a more serious condition.

In conclusion, this evolving collaborative network allowed the collection of detailed data in one of the largest studies from a LMIC region to provide a first description of associated factors for hospital or ICU admission in developing countries. The demographic, clinical and laboratory parameters that were identified could help care providers in different settings (outpatient clinic, emergency room or general pediatric wards) to identify children at higher risk for a complicated disease course, and direct policy makers to prioritize pediatric subgroups for prevention and treatment strategies.

## Data Availability Statement

The raw data supporting the conclusions of this article will be made available by the authors, without undue reservation.

## Ethics Statement

The studies involving human participants were reviewed and the registry and study protocol were reviewed and approved by the local Institutional Review Board of each participant center. The research was deemed of minimal risk because data was collected for routine clinical practice and the requirement for informed consent was waived. Written informed consent from the participants' legal guardian/next of kin was not required to participate in this study in accordance with the national legislation and the institutional requirements.

## Author Contributions

EL-M and EA: study conception and design. EL-M, GC-M, MB, DD, JT, RU-G, PL, RD, PP, JP, XN, CM, ML, GE, CD, KL, PQ, MR, JR-A, AE-V, MC, OM, EB, JC, AM, AJ-Z, LD, MM, and NG: data collection. EL-M, EC, and EA: analysis and interpretation of results. E-LM and EA: draft manuscript preparation. All authors reviewed the results and approved the final version of the manuscript.

## Funding

The Research Electronic Data Capture (REDCap) database used for this study was supported by the Grant Number UL1 TR002535, from NIH/NCATS Colorado CTSA.

## Conflict of Interest

The authors declare that the research was conducted in the absence of any commercial or financial relationships that could be construed as a potential conflict of interest.

## Publisher's Note

All claims expressed in this article are solely those of the authors and do not necessarily represent those of their affiliated organizations, or those of the publisher, the editors and the reviewers. Any product that may be evaluated in this article, or claim that may be made by its manufacturer, is not guaranteed or endorsed by the publisher.
