# Supplementary material for: Factors Associated With Hospitalization or Intensive Care Admission in Children With COVID-19 in Latin America
Source: Front Pediatr. 2022 Apr 14;10:868297. doi: 10.3389/fped.2022.868297 (PMC9048675; doi:10.3389/fped.2022.868297)
Supplement: Supplementary file 1 [file Data_Sheet_1.pdf]

Supplementary Table 1
Proportion of patients with specific clinical presentations according to age groups.

	No respiratory symptoms or fever*	Diarrhea without respiratory symptoms*
All patients, n=1063 (n, %)	104 (9.8%)	96 (9.0%)
< 1 year, n=241	13 (5.4%)	19 (7.9%)
1-5 years, n=386	39 (10.1%)	52 (13.5%)
6-9 years, n=176	14 (7.9%)	11 (6.2%)
>10 years, n=260	38 (14.6%)	14 (5.4%)

*P<0.01 for the age distribution

Supplementary Table 2.
Frequency of missing data for each variable

			Hospitalized	
**Characteristic**	**All patients (n=1063)**	**OutPatient care (n=563)**	**General Ward (n=419)**	**PICU (n=81)**
**Age**	0 (0.0)	0 (0.0)	0 (0.0)	0 (0.0)
**Sex**	0 (0.0)	0 (0.0)	0 (0.0)	0 (0.0)
**Race or ethnic group**[a]	76 (7.1)	46 (8.2)	23 (5.5)	7 (8.6)
**Population**	183 (17.2)	148 (26.3)	34 (8.1)	1 (1.2)
**Level of education of caregiver**	815 (76.7)	448 (79.6)	309 (73.7)	58 (71.6)
**Full immunization coverage**	204 (19.2)	86 (15.3)	109 (26.0)	9 (11.1)
**BMI**[b]	501 (47.1)	304 (54.0)	177 (42.2)	20 (24.7)
**Co-morbidities**	0 (0.0)	0 (0.0)	0 (0.0)	0 (0.0)
**Symptoms or Signs described or present on initial evaluation**				
Anosmia	2 (0.2)	0 (0.0)	2 (0.5)	0 (0.0)
Dysgeusia	2 (0.2)	0 (0.0)	2 (0.5)	0 (0.0)
Skin rash	4 (0.4)	1 (0.2)	3 (0.7)	0 (0.0)
Conjunctivitis	4 (0.4)	1 (0.2)	3 (0.7)	0 (0.0)
Abdominal pain	52 (4.9)	20 (3.5)	25 (5.9)	7 (8.6)
Fever	1 (0.1)	0 (0.0)	1 (0.2)	0 (0.0)
Cough	2 (0.2)	0 (0.0)	2 (0.5)	0 (0.0)
Pharyngitis	4 (0.4)	0 (0.0)	4 (0.9)	0 (0.0)
Rhinitis	3 (0.3)	0 (0.0)	3 (0.7)	0 (0.0)
Headache	5 (0.5)	0 (0.0)	5 (1.2)	0 (0.0)
Myalgia	8 (0.7)	2 (0.4)	6 (1.4)	0 (0.0)
Malaise	5 (0.5)	1 (0.2)	4 (0.9)	0 (0.0)
Diarrhea	4 (0.4)	0 (0.0)	4 (0.9)	0 (0.0)
Vomit	4 (0.4)	0 (0.0)	4 (0.9)	0 (0.0)
Dyspnea	3 (0.3)	2 (0.4)	1 (0.2)	0 (0.0)
Hypoxia	3 (0.3)	2 (0.4)	1 (0.2)	0 (0.0)
Hemoptisis	5 (0.5)	2 (0.4)	3 (0.7)	0 (0.0)
Altered mental status	5 (0.5)	2 (0.4)	3 (0.7)	0 (0.0)
Seizures	5 (0.5)	2 (0.4)	2 (0.5)	1 (1.2)
Dehydration	8 (0.7)	3 (0.5)	4 (0.9)	1 (1.2)

Shock	6 (0.6)	4 (0.7)	2 (0.5)	0 (0.0)
**Onset of symptoms to, median (IQR)**	42 (3.9)	9 (1.6)	33 (7.9)	0 (0.0)
**Laboratory values**				
Hemoglobin	485 (45.6)	434 (77.1)	47 (11.2)	4 (4.9)
Platelets	466 (43.8)	434 (77.1)	28 (6.7)	4 (4.9)
White blood cells	457 (43.0)	432 (76.7)	22 (5.2)	3 (3.7)
Neutrophils	460 (43.3)	433 (76.9)	22 (5.2)	5 (6.2)
Lymphocytes	463 (43.6)	433 (76.9)	25 (6.0)	5 (6.2)
Neutrophil to lymphocyte ratio	464 (43.6)	433 (76.9)	26 (6.2)	5 (6.2)
C-reactive protein	547 (51.5)	449 (79.7)	87 (20.8)	11 (6.2)
**Chest radiograph**	503 (43.3)	387 (68.7)	106 (25.2)	10 (12.3)

Abbreviations: PICU, pediatric intensive care unit; BMI, body mass index (calculated as weight in kilograms divided by height in meters squared); IQR, interquartile range;

[a] Race/ethnic group was collected by study personnel based on auto reporting by the study participants. “Mixed race” refers to an individual of mixed European/Native heritage.

[b] BMI, Body mass index was calculated as weight in kilograms divided by height in meters squared

Supplementary Table 3.
Characteristics of Hospital Admission Among the 500 Hospitalized Children

	**General Ward (n=419)**	**PICU (n=81)**
**Hospital length of stay in days,** Median (IQR)	5 (3 to 7)	12 (8 to 20.1)
**PICU length of stay in days,** Median (IQR)	-	5 (2 to 10)
**Intubation, No. (%)**	-	31 (38.3)
**Intubation duration in days,** Median (IQR)	-	5 (3 to 10)
**Oxygen, No. (%)**	151 (36.0)	70 (86.4)
**Oxygen duration in days,** Median (IQR)	3 (2 to 5)	7 (4 to 17)

Abbreviations: PICU, Pediatric intensive care unit

Supplementary Table 4.

Demographic and clinical characteristics associated with hospitalization (general ward and intensive care) among all patients (analysis using the complete dataset including missing data), and patients included in cities that used an active surveillance system (analysis using multiple imputation chained equations for missing data).

	All patients. Complete Dataset, including missing data				Patients from Active Surveillance Sites. Multiple Imputation			
**Characteristic**	**Outpatient care (n=563)**	**Hospitalization (n=500)**	**Univariate analysis OR (95% CI)**	**Multivariable analysis OR (95% CI)**	**Outpatient care (n=526)**	**Hospitalization (n=319)**	**Univariate analysis OR (95% CI)**	**Multivariable analysis OR (95% CI)**
**Age groups, y No. (%)**								
<1	109 (19.4)	132 (26.4)	1.42 (0.86 to 2.34)	1.78 (0.98 to 3.21)	100 (19.0)	70 (21.9)	1.16 (0.84 to 1.59)	2.11 (1.25 to 3.55)
1-5	214 (38.0)	172 (344.4)	0.94 (0.75 to 1.18)	1.15 (0.92 to 1.44)	204 (38.8)	117 (36.7)	0.95 (0.69 to 1.31)	1.43 (1.02 to 1.99)
6-10	95 (16.9)	81 (16.2)	Ref.	Ref.	91 (17.3)	55 (17.2)	Ref.	Ref.
≥10	145 (25.7)	115 (23.0)	0.93 (0.66 to 1.31)	0.91 (0.51 to 1.62)	131 (24.9)	77 (24.1)	0.97 (0.69 to 1.37)	1.07 (0.49 to 2.31)
**Sex, No. (%)**								
Male	291 (51.7)	279 (55.8)	Ref.	-	270 (51.3)	183 (57.4)	Ref.	-
Female	272 (48.3)	221 (44.2)	0.85 (0.68 to 1.06)	-	256 (48.7)	136 (42.6)	0.78 (0.59 to 1.03)	-
**Race or ethnic group[a], No. (%)**								
Caucasian	285 (55.1)	174 (37.0)	Ref.	Ref.	283 (58.7)	164 (54.3)	Ref.	-
Native	3 (0.6)	13 (2.8)	7.09 (2.3 to 21.64)	6.27 (2.59 to 15.19)	0 (0.0)	3 (1.0)	-	-
Black or African American	6 (1.2)	3 (0.6)	0.82 (0.10 to 6.87)	0.86 (0.14 to 5.32)	6 (1.2)	1 (0.3)	0.29 (0.03 to 3.62)	-
Mixed race	223 (43.1)	280 (59.6)	2.06 (0.54 to 7.83)	1.87 (0.55 to 6.37)	193 (40.0)	134 (44.4)	1.20 (0.48 to 2.98)	-
**Population, No. (%)**								
Urban	393 (94.7)	414 (89.0)	Ref.	-	362 (95.8)	267 (93.7)	Ref.	-
Rural	22 (5.3)	51 (11.0)	2.20 (1.42 to 3.40)	-	16 (4.2)	18 (6.3)	1.32 (0.69 to 2.53)	-
**Full immunization coverage, No. (%)**								
Yes	435 (91.2)	335 (87.7)	0.69 (0.31 to 1.54)	-	420 (91.1)	249 (90.9)	0.85 (0.45 to 1.63	-
**Nutritional status[b] No. (%)**								
Thinness	17 (6.6)	48 (15.8)	2.67 (2.00 to 3.56)	-	16 (6.5)	32 (16.7)	1.58 (0.78 to 3.19)	-
Normal Weight	173 (66.8)	183 (60.4)	Ref.	-	167 (67.9)	116 (60.7)	Ref.	-
Overweight	47 (18.1)	36 (11.9)	0.72 (0.48 to 1.11)	-	41 (16.7)	19 (9.9)	0.80 (0.44 to 1.46)	-
Obese	22 (8.5)	36 (11.9)	1.55 (0.90 to 2.67)	-	22 (8.9)	24 (12.6)	1.17 (0.69 to 1.97)	-
**Co-morbidities- No. (%)**								
Chronic lung disease	54 (9.6)	69 (13.8)	1.51 (0.75 to 3.04)	-	50 (9.5)	63 (19.7)	2.34 (1.44 to 3.80)	**3.33 (1.83 to 6.09)**
Congenital heart disease	6 (1.1)	12 (2.40)	2.28 (0.55 to 9.51)	-	5 (0.9)	5 (1.6)	1.66 (0.2 to 12.83)	-
Chronic gastrointestinal disease	2 (0.4)	7 (1.40)	3.98 (1.40 to 11.31)	3.21 (1.21 to 8.53)	2 (0.4)	4 (1.2)	3.33 (1.29 to 8.56)	-

Neurologic disease	7 (1.2)	52 (10.4)	9.22 (3.8 to 22.34)	10.85 (4.25 to 27.72)	6 (1.1)	42 (13.2)	13.14 (6.53 to 26.44)	16.97 (7.57 to 38.05)
Metabolic or endocrine disorder	1 (0.2)	7 (1.40)	7.98 (0.99 to 64.09)	10.22 (1.71 to 61.01)	1 (0.2)	4 (1.2)	6.67 (0.82 to 54.59)	10.95 (1.77 to 67.81)
Immune deficiency	13 (2.3)	75 (15.0)	7.48 (2.78 to 20.1)	8.89 (2.80 to 28.20)	8 (1.5)	54 (16.9)	13.19 (6.3 to 27.5)	21.44 (11.08 to 41.46)
Preterm birth	9 (1.6)	24 (4.8)	3.10 (1.58 to 6.10)	2.59 (1.30 to 5.17)	9 (1.7)	21 (6.6)	4.05 (2.35 to 6.97)	2.99 (1.75 to 5.11)

Abbreviations: OR, odds ratio; IQR, interquartile range

[a] Race/ethnic group was collected by study personnel based on auto reporting by the study participants. "Mixed race" refers to an individual of mixed European/Native heritage [b] Nutritional status was classified based on standard deviations for body mass index according to the World Health Organization into thinness (below -2 standard deviations [SD]), normal nutritional status (between -2 and +1 SD), overweight (between +1 and +2 SD) and obesity (above +2 SD). Body mass index was calculated as weight in kilograms divided by height in meters squared

Supplementary Table 5.

Presenting signs and symptoms associated with hospitalization (general ward and intensive care) among all patients (analysis using the complete dataset including missing data), and patients included in cities that used an active surveillance system (analysis using multiple imputation chained equations for missing data).

Symptoms or Signs described or present on admission- No. (%)	All patients. Complete Dataset, including missing data				Patients from Active Surveillance Sites. Multiple Imputation			
	OutPatient care (n=563)	Hospitalization (n=500)	Univariate analysis OR (95% CI)	Multivariable analysis OR (95% CI)	OutPatient care (n=526)	Hospitalization (n=319)	Univariate analysis OR (95% CI)	Multivariable analysis OR (95% CI)
Anosmia	19 (4.7)	6 (1.8)	0.36 (0.13 to 1.02)	-	17 (4.5)	3 (1.3)	-	-
Dysgeusia	19 (4.7)	8 (2.3)	0.48 (0.21 to 1.08)	-	16 (4.2)	4 (1.7)	-	-
Skin rash	11 (2.0)	23 (4.6)	2.43 (1.41 to 4.19)	2.72 (1.48 to 4.97)	11 (2.1)	11 (3.4)	1.67 (0.79 to 3.50)	2.53 (1.01 to 6.32)
Conjunctivitis	8 (1.4)	7 (1.4)	0.99 (0.47 to 2.07)	-	6 (1.1)	2 (0.6)	0.54 (0.19 to 1.52)	-
Abdominal pain	62 (11.4)	65 (13.9)	1.25 (0.94 to 1.66)	-	59 (11.7)	44 (14.4)	1.24 (0.97 to 1.60)	2.48 (1.46 to 4.21)
Fever	424 (75.3)	321 (64.3)	0.59 (0.29 to 1.21)	-	407 (77.4)	221 (69.5)	0.66 (0.33 to 1.35)	-
Cough	324 (57.5)	256 (51.4)	0.78 (0.46 to 1.32)	-	307 (58.4)	178 (56.1)	0.91 (0.41 to 1.99)	-
Pharyngitis	177 (31.4)	71 (14.3)	0.36 (0.29 to 0.45)	0.33 (0.23 to 0.46)	172 (32.7)	50 (15.8)	0.38 (0.32 to 0.47)	0.42 (0.29 to 0.60)
Rhinitis	181 (32.1)	133 (26.8)	0.77 (0.31 to 1.90)	-	166 (31.6)	94 (29.6)	0.91 (0.39 to 2.15)	-
Headache	114 (20.2)	48 (9.7)	0.42 (0.23 to 0.77)	-	106 (20.1)	28 (8.8)	0.38 (0.18 to 0.82)	-
Myalgia	60 (10.7)	30 (6.1)	0.54 (0.27 to 1.07)	0.52 (0.29 to 0.94)	56 (10.7)	19 (6.0)	0.53 (0.26 to 1.08)	0.51 (0.30 to 0.88)
Malaise	182 (32.4)	159 (32.1)	0.98 (0.47 to 2.07)	-	172 (32.8)	101 (31.9)	0.96 (0.47 to 1.93)	-
Diarrhea	139 (24.7)	80 (16.1)	0.59 (0.33 to 1.04)	0.42 (0.25 to 0.70)	131 (24.9)	46 (14.5)	0.51 (0.23 to 1.11)	0.34 (0.17 to 0.69)
Vomit	107 (19.0)	92 (18.5)	0.97 (0.55 to 1.70)	-	101 (19.2)	59 (18.7)	0.96 (0.52 to 1.76)	-
Dyspnea	41 (7.3)	190 (38.1)	7.80 (4.61 to 13.19)	-	37 (7.1)	137 (43.1)	9.94 (7.01 to 14.09)	-
Hypoxia	8 (1.4)	167 (33.5)	34.77 (7.6 to 159.6)	34.88 (7.56 to 160.8)	5 (0.9)	134 (42.1)	74.9 (9.66 to 581.43)	80.79 (10.22 to 638.-97)
Altered mental status	4 (0.7)	39 (7.8)	11.85 (3.57 to 39.33)	-	3 (0.6)	24 (7.6)	13.96 (4.26 to 45.77)	-
Seizures	8 (1.4)	38 (7.6)	5.72 (1.73 to 18.93)	5.27 (1.49 to 18.62)	6 (1.1)	30 (9.4)	8.93 (2.85 to 28.03)	11.46 (6.35 to 20.69)
Dehydration	7 (1.2)	60 (12.1)	10.89 (3.88 to 30.58)	16.54 (4.51 to 60.68)	7 (1.3)	27 (8.5)	6.78 (3.34 to 13.76)	9.47 (3.08. to 29.12)
Shock	0	27 (5.4)	-	-	0	16 (5.0)	-	-
**Onset of symptoms to, median (IQR)**	2 (1-3)	1 (0-3)	1.01 (0.96 to 1.05)	-	2 (1 to 3)	1 (0 to 3)	0.99 (0.96 to 1.04)	-

Supplementary Table 6.

Demographic and clinical characteristics associated with intensive care admission among all patients (analysis using the complete dataset including missing data), and patients included in cities that used an active surveillance system (analysis using multiple imputation chained equations for missing data).

	All patients. Complete Dataset, including missing data				Patients from Active Surveillance Sites. Multiple Imputation			
**Characteristics**	**General Ward (n=419)**	**Pediatric intensive care unit (n=81)**	**Univariate analysis OR (95% CI)**	**Multivariable analysis OR (95% CI)**	**General Ward (n=265)**	**Pediatric Intensive Care Unit (n=54)**	**Univariable analysis OR (95% CI)**	**Multivariable analysis OR (95% CI)**
**Age groups, y No. (%)**								
<1	113 (27.0)	19 (23.5)	0.80 (0.53. to 1.22)	-	56 (21.1)	14 (25.9)	1.12 (0.87 to 1.45)	-
1-5	141 (33.6)	31 (38.3)	1.05 (0.66 to 1.66)	-	99 (37.4)	18 (33.3)	0.82 (0.61 to 1.09)	-
6-9	67 (16.0)	14 (17.3)	Ref.	-	45 (17.0)	10 (18.5)	Ref.	-
≥10	98 (23.4)	17 (21.0)	0.83 (0.47 to 1.46)	-	65 (24.5)	12 (22.2)	0.83 (0.39 to 1.74)	-
**Sex, No. (%)**								
Male	237 (56.6)	42 (51.9)	Ref.	-	151 (57.0)	32 (59.3)	Ref.	-
Female	182 (43.4)	39 (48.1)	1.21 (0.73 to 2.01)	-	114 (43.0)	22 (40.7)	0.91 (0.76 to 1.08)	-
**Race or ethnic group[a], No. (%)**								
Caucasian	149 (37.6)	25 (33.8)	Ref.	-	144 (56.7)	20 (41.7)	Ref.	-
Native	11 (2.8)	2 (2.7)	1.08 (0.27 to 4.36)	-	2 (0.8)	1 (2.1)	4.18 (0.45 to 38.52)	-
Black or African American	3 (0.8)	0 (0.0)	-	-	1 (0.4)	0 (0.0)	-	-
Mixed race	233 (58.8)	47 (63.5)	1.20 (0.36 to 4.05)	-	107 (42.1)	27 (56.2)	1.94 (0.36 to 10.40)	-
**Population, No. (%)**								
Urban	347 (90.1)	67 (83.8)	Ref.	Ref.	219 (94.4)	48 (90.6)	Ref.	-
Rural	38 (9.9)	13 (16.2)	1.77 (1.06 to 2.95)	1.73 (1.05 to 2.86)	13 (5.6)	5 (9.4)	1.59 (0.74 to 3.40)	-
**Full immunization coverage, No. (%)**								
Yes	274 (88.4)	61 (84.7)	0.73 (0.44 to 1.22)	-	207 (91.2)	42 (89.4)	0.81 (0.33 to 1.96)	-
**Nutritional Status[b], No. (%)**								
Thinness	37 (15.3)	11 (18.0)	1.30 (0.92 to 1.84)	-	26 (17.1)	6 (15.4)	1.17 (0.62 to 2.20)	-
Normal Weight	149 (61.6)	34 (55.8)	Ref.	-	95 (62.5)	21 (53.9)	Ref.	-
Overweight	31 (12.8)	5 (8.2)	0.71 (0.21 to 2.34)	-	15 (9.9)	4 (10.3)	0.92 (0.38 to 2.22)	-
Obese	25 (10.33)	11 (18.0)	1.93 (0.81 to 4.59)	-	16 (10.5)	8 (20.5)	1.44 (0.67 to 3.09)	-
**Co-morbidities- No. (%)**								
Chronic lung disease	58 (13.8)	11 (13.6)	0.98 (0.25 to 3.91)	-	53 (20.0)	10 (18.5)	0.91 (0.22 to 3.79)	-
Congenital heart disease	9 (2.1)	3 (3.7)	1.75 (0.54 to 5.69)	-	4 (1.5)	1 (1.8)	1.23 (0.12 to 12.05)	-
Chronic gastrointestinal disease	6 (1.4)	1 (1.2)	0.86 (0.73 to 10.18)	-	4 (1.5)	0 (0.0)	-	-
Neurologic disease	41 (9.8)	11 (13.6)	1.45 (0.74 to 2.83)	-	33 (12.4)	9 (16.7)	1.41 (0.70 to 2.80)	-

Metabolic or endocrine disorder	4 (0.9)	3 (3.7)	3.99 (1.35 to 11.76)	5.19 (1.97 to 13.62)	3 (1.1)	1 (1.8)	1.65 (0.63 to 4.28)	-
Immune deficiency	57 (13.6)	18 (22.2)	1.81 (0.96 to 3.43)	1.83 (1.02 to 3.28)	42 (15.8)	12 (22.2)	1.52 (0.83 to 2.78)	1.91 (1.03 to 3.53)
Preterm birth	17 (4.1)	7 (8.6)	2.24 (1.26 to 3.95)	2.33 (1.30 to 4.19)	14 (5.3)	7 (13.0)	2.67 (1.41 to 5.05)	2.45 (1.35 to 4.42)

Abbreviations: OR, odds ratio; IQR, interquartile range; WBC, white blood cell count

[a] Race/ethnic group was collected by study personnel based on auto reporting by the study participants. “Mixed race” refers to an individual of mixed European/Native heritage. [b] Nutritional status was classified based on standard deviations for body mass index according to the World Health Organization into thinness (below -2 standard deviations [SD]), normal nutritional status (between -2 and +1 SD), overweight (between +1 and +2 SD) and obesity (above +2 SD). Body mass index was calculated as weight in kilograms divided by height in meters squared. [c] Values < 11 g/dL. [d] platelets <150,000 x 10^3 cells/uL.

* Number of patients with available data

Supplementary Table 7.

Presenting signs/symptoms, laboratory values and radiographic findings associated with intensive care admission among all patients (analysis using the complete dataset including missing data), and patients included in cities that used an active surveillance system (analysis using multiple imputation chained equations for missing data).

Symptoms or Signs described or present on admission- No. (%)	All patients. Complete Dataset, including missing data				Patients from Active Surveillance Sites. Multiple Imputation			
	General Ward (n=419)	Pediatric intensive care unit (n=81)	Univariate analysis OR (95% CI)	Multivariable analysis OR (95% CI)	General Ward (n=265)	Pediatric Intensive Care Unit (n=54)	Univariable analysis OR (95% CI)	Multivariable analysis OR (95% CI)
Anosmia	3 (1.1)	3 (5.1)	5.00 (1.06 to 23.49)	-	2 (1.0)	1 (2.3)	-	-
Dysgeusia	6 (2.1)	2 (3.3)	1.63 (0.12 to 21.91)	-	4 (2.0)	0 (0.0)	-	-
Skin rash	21 (5.0)	2 (2.5)	0.48 (0.15 to 1.52)	-	10 (3.8)	1 (1.8)	0.48 (0.13 to 1.81)	-
Conjunctivitis	6 (1.4)	1 (1.2)	0.85 (0.07 to 10.75)	-	2 (0.7)	0 (0.0)	-	-
Abdominal pain	54 (13.7)	11 (14.9)	1.10 (0.39 to 3.11)	-	40 (15.6)	4 (8.2)	0.46 (0.29 to 0.70)	-
Fever	263 (62.9)	58 (71.6)	1.49 (1.06 to 2.08)	-	179 (67.8)	42 (77.8)	1.66 (1.44 to 1.92)	-
Cough	209 (50.1)	47 (58.0)	1.37 (0.74 to 2.54)	-	149 (56.6)	29 (53.7)	0.89 (0.43 to 1.86)	-
Pharyngitis	59 (14.2)	12 (14.8)	1.05 (0.36 to 3.01)	-	43 (16.3)	7 (13.0)	0.76 (0.22 to 2.62)	-
Rhinitis	111 (26.7)	22 (27.2)	1.02 (0.44 to 2.40)	-	80 (30.4)	14 (25.9)	0.80 (0.26 to 2.45)	-
Headache	39 (9.4)	9 (11.1)	1.20 (0.63 to 2.29)	-	23 (8.7)	5 (9.3)	1.07 (0.48 to 2.35)	-
Myalgia	22 (5.3)	8 (9.9)	1.95 (0.51 to 7.38)	-	15 (5.7)	4 (7.4)	1.32 (0.21 to 8.42)	-
Malaise	123 (29.6)	36 (44.4)	1.90 (0.76 to 4.76)	-	81 (30.8)	20 (37.0)	1.32 (0.52 to 3.37)	
Diarrhea	61 (14.7)	19 (23.5)	1.78 (1.12 to 2.82)	2.12 81.09 to 4.14)	34 (12.9)	12 (22.2)	1.93 (1.16 to 3.21)	3.99 (2.59 to 6.13)
Vomit	77 (18.6)	15 (18.5)	1.00 (0.58 to 1.70)	-	51 (19.5)	8 (14.8)	0.73 (0.54 to 0.97)	0.30 (0.16 to 0.57)
Dyspnea	137 (32.8)	53 (65.4)	3.88 (1.83 to 8.24)	-	99 (37.5)	38 (70.4)	3.98 (1.55 to 10.18)	2.96 (1.46 to 5.98)
Hypoxia	115 (27.5)	52 (64.2)	4.72 (2.07 to 10.79)	4.52 (1.36 to 15.03)	95 (36.0)	39 (72.2)	4.64 (1.96 to 11.08)	3.72 (1.89 to 7.32)
Altered mental status	13 (3.1)	26 (32.1)	14.65 (5.0 to 42.90)	3.50 (0.70 to 17.48)	9 (3.4)	15 (27.8)	10.85 (3.4 to 34.67)	-
Seizures	21 (5.0)	17 (21.2)	5.09 (3.33 to 7.76)	2.57 (1.16 to 5.69)	17 (6.4)	13 (24.1)	4.61 (3.16 to 6.74)	8.99 (4.34 to 18.60)
Dehydration	41 (9.9)	19 (23.7)	2.84 (1.12 to 7.20)	-	20 (7.6)	7 (13.0)	1.82 (0.79 to 4.19)	-
Shock	5 (1.2)	22 (27.2)	30.72 (13.0 to 72.4)	11.08 (3.1 to 39.82)	2 (0.8)	14 (25.9)	44.38 (8.8 to 224.8)	35.88 (2.8 to 464.3)
**Onset of symptoms to, median (IQR)**	1 (0 to 3)	1 (0 to 3)	0.98 (0.91 to 1.06)	-				
**Laboratory values**					1 (1 to 3)	1 (0 to 3)	1.00 (0.94 to 1.06)	
Hemoglobin (g/dL)								
No.*	372	77			219	51		
Median (IQR)	12.3 (10.9 to 13.5)	11.4 (9.5 to 13.3)	0.89 (0.81 to 0.99)	-	12.5 (11.1 to 13.8)	12.0 (9.8 to 14.2)	0.95 (0.87 to 1.03)	
Anemia[c]	106 (28.5)	34 (44.2)	1.98 (1.20 to 3.28)	2.58 (1.29 to 5.17)	52 (23.7)	20 (39.2)	2.07 (1.13 to 3.78)	2.04 (1.41 to 2.95)
Platelets (10^3 cells/uL)								
No.*	391	77			239	51		
Median (IQR)	299 (218 to 386)	276 (158 to 374.5)	0.99 (0.997 to 1.0)	-	292.0 (210 to 377)	277.0 (137 to 334)	0.998 (0.995 to 1.0)	-
Thrombocytopenia[d]	42 (10.7)	18 (23.4)	2.53 (1.54 to 4.18)		27 (11.3)	14 (27.4)	2.87 (1.34 to 6.14)	
WBC (10^3 cells/uL)								
No.*	397	78			246	51		
Median (IQR)	10.0 (6.8 to 15.0)	12.2 (6.5 to 14.9)	1.00 (0.97 to 1.04)	-	9.9 (6.7 to 14.9)	10.6 (5.7 to 14.2)	0.982 (0.93 to 1.04)	-
Neutrophils (10^3 cells/uL)				-				
No.*	397	76			245	50		
Median (IQR)	5.2 (2.5 to 9.3)	6.0 (3.0 to 9.3)	0.99 (0.95 to 1.05)		5.5 (2.5 to 9.5)	4.9 (1.7 to 7.9)	0.968 (0.89 to 1.05)	-
Lymphocytes (10^3 cells/uL)				-				

No.*	394	76			243	50		
Median (IQR)	2.8 (1.6 to 4.6)	3.0 (0.9 to 5.2)	1.03 (0.96 to 1.10)		2.6 (1.3 to 4.1)	3.1 (0.7 to 5.6)	1.03 (1.01 to 1.05)	-
Neutrophil to lymphocyte ratio				-				
No.*	393	76			242	50		
Median (IQR)	1.8 (0.7 to 4.3)	1.8 (0.8 to 4.8)	1.01 (0.95 to 1.08)		2.1 (0.8 to 5.0)	1.4 (0.6 to 4.3)	0.972 (0.92 to 1.02)	
Values > 5	86 (21.9)	18 (23.7)	1.11 (0.63 to 1.96)		60 (24.8)	11 (22.0)	0.87 (0.47 to 1.58)	-
C-reactive protein (mg/dL)				-				
No.*	332	70			216	48		
Median (IQR)	12.9 (4.2 to 39.5)	18.9 (7.0 to 102.7)	1.04 (1.02 to 1.05)		16.5 (9.0 to 48.0)	22.3 (10.0 to 100.9)	1.04 (1.02 to 1.06)	
Values > 50 mg/dL	70 (21.1)	23 (32.9)	1.83 (1.04 to 3.22)		53 (24.5)	17 (35.4)	1.76 (1.04 to 2.96)	-
**Chest radiograph performed, No. (%)**				-				
Yes	313 (74.7)	71 (87.65)			210	47		
Normal	194 (62.0)	29 (40.8)	Ref.	Ref.	141 (67.1)	20 (42.6)	Ref.	Ref.
Bronchial wall thickening	28 (8.9)	10 (14.1)	2.39 (0.93 to 6.16)	2.89 (1.21 to 6.92)	18 (8.6)	9 (19.1)	3.09 (1.36 to 7.05)	3.61(1.63 to 7.98)
Interstitial infiltrates	55 (17.6)	16 (22.5)	1.94 (1.02 to 3.69)	1.08 (0.52 to 2.28)	27 (12.9)	9 (19.1)	2.21 (0.97 to 4.99)	1.49 (0.56 to 3.99)
Consolidation	23 (7.3)	8 (11.3)	2.33 (1.19 to 4.53)	1.12 (0.49 to 2.56)	20 (9.5)	6 (12.8)	2.15 (1.21 to 3.83)	1.11 (0.60 to 2.05)
Interstitial infiltrates + consolidation	13 (4.1)	8 (11.3)	4.12 (1.11 to 15.3)	1.15 (0.24 to 5.45)	4 (1.9)	3 (6.4)	4.19 (0.81 to 21.70)	1.28 (0.18 to 9.18)

Supplementary Table 8.

Risk factors associated with supplementary oxygen requirement among pediatric patients with COVID-19.

			Complete Data		Multiple Imputation	
**Characteristic**	**Non-oxygen (n=836)**	**Oxygen (n=227)**	**Univariable analysis OR (95% CI)**	**Multivariable analysis OR (95% CI)**	**Univariable analysis OR (95% CI)**	**Multivariable analysis OR (95% CI)**
**Age groups, y No. (%)**						
<1	182 (21.8)	59 (26.0)	1.14 (0.85 to 1.53)	2.09 (1.23 to 3.53)	1.14 (0.85 to 1.53)	2.09 (1.23 to 3.53)
1-5	306 (36.6)	80 (35.2)	0.92 (0.72 to 1.16)	1.19 (0.81 to 1.73)	0.92 (0.72 to 1.16)	1.19 (0.81 to 1.73)
6-9	137 (16.4)	39 (17.2)	Ref.	Ref.	Ref.	Ref.
≥10	211 (25.2)	49 (21.6)	0.81 (0.50 to 1.32)	0.89 (0.45 to 1.77)	0.81 (0.50 to 1.32)	0.89 (0.45 to 1.77)
**Sex, No. (%)**						
Male	433 (51.8)	137 (60.3)	Ref.	-	Ref.	-
Female	403 (48.2)	90 (39.6)	0.70 (0.50 to 1.00)	-	0.70 (0.50 to 1.00)	-
**Race or ethnic group, No. (%)**						
Caucasian	359 (47.6)	90 (42.6)	Ref.	-	Ref.	-
Native	11 (1.4)	5 (2.4)	1.86 (0.84 to 4.15)	-	1.87 (0.83 to 4.19)	-
Black or African American	9 (1.2)	0 (0.0)	-	-	-	-
Mixed race	387 (49.9)	116 (55.0)	1.23 (0.66 to 2.28)	-	1.23 (0.71 to 2.14)	-
**Population, No. (%)**						
Urban	622 (92.1)	185 (90.2)	Ref.	-	Ref.	-
Rural	53 (7.8)	20 (9.8)	1.27 (0.78 to 2.06)	-	1.26 (0.76 to 2.10)	-
**Full immunization coverage, No. (%)**						
Yes	610 (90.1)	160 (87.9)	0.80 (0.45 to 1.41)	-	0.78 (0.44 to 1.37)	-
**BMI (continuous), No. (%)**				-		-
Thinness	36 (8.4)	29 (21.3)	2.87 (1.82 to 4.51)	-	1.70 (0.86 to 3.39)	-
Normal Weight	278 (65.3)	78 (57.3)	Ref.	-	Ref.	-
Overweight	70 (16.4)	13 (9.6)	0.66 (0.43 to 1.02)	-	0.81 (0.50 to 1.32)	-
Obese	42 (9.9)	16 (11.8)	1.36 (0.69 to 2.76)	-	1.16 (0.66 to 2.03)	-
**Co-morbidities- No. (%)**						
Chronic lung disease	70 (8.4)	53 (23.3)	3.33 (2.05 to 5.41)	4.45 (2.35 to 8.41)	3.33 (2.05 to 5.41)	4.45 (2.35 to 8.41)
Congenital heart disease	12 (1.4)	6 (2.6)	1.86 (0.49 to 7.07)	-	1.86 (0.49 to 7.01)	-
Chronic gastrointestinal disease	6 (0.7)	3 (1.3)	1.85 (0.41 to 8.39)	-	1.85 (0.41 to 8.39)	-
Neurologic disease	29 (3.5)	30 (13.2)	4.24 (2.78 to 6.46)	5.21 (3.30 to 8.24)	4.24 (7.78 to 6.46)	5.21 (3.30 to 8.24)
Metabolic or endocrine disorder	5 (0.6)	3 (1.3)	2.22 (0.50 to 9.91)	-	2.22 (0.50 to 9.91)	-
Immune deficiency	52 (6.2)	36 (15.9)	2.84 (2.09 to 3.85)	4.44 (2.56 to 7.69)	2.84 (2.09 to 3.85)	4.44 (2.56 to 7.69)
Preterm birth	18 (2.1)	15 (6.6)	3.21 (1.50 to 6.87)	-	3.21 (1.50 to 6.88)	-
**Symptoms or Signs described or present on admission- No. (%)**						
Anosmia	22 (3.7)	3 (2.0)	0.54 (0.12 to 2.49)	-	-	-
Dysgeusia	23 (3.8)	4 (2.7)	0.69 (0.18 to 2.66)	-	-	-
Skin rash	28 (3.4)	6 (2.7)	0.79 (0.39 to 1.61)	-	0.79 (0.38 to 1.61)	-
Conjunctivitis	12 (1.4)	3 (1.3)	0.92 (0.44 to 1.95)	-	0.96 (0.43 to 2.13)	-
Abdominal pain	115 (14.4)	12 (5.6)	0.35 (0.12 to 0.99)	-	0.36 (0.13 to 0.99)	-
Fever	596 (71.4)	149 (65.6)	0.77 (0.33 to 1.77)	-	0.77 (0.33 to 1.77)	-

Cough	419 (50.2)	161 (71.2)	2.46 (1.02 to 5.93)	-	2.46 (1.02 to 5.92)	-
Pharyngitis	214 (25.7)	34 (15.1)	0.51 (0.35 to 0.76)	0.68 (0.46 to 0.99)	0.51 (0.35 to 0.75)	0.64 (0.46 to 0.90)
Rhinitis	247 (29.6)	67 (29.6)	1.00 (0.56 to 1.78)	-	1.01 (0.57 to 1.79)	-
Headache	146 (17.5)	16 (7.1)	0.36 (0.19 to 0.70)	-	0.36 (0.18 to 0.69)	-
Myalgia	72 (8.7)	18 (8.0)	0.92 (0.37 to 2.27)	-	0.91 (0.37 to 2.26)	-
Malaise	268 (32.2)	73 (32.4)	1.01 (0.67 to 1.54)	-	1.01 (0.66 to 1.54)	-
Diarrhea	194 (23.3)	25 (11.1)	0.41 (0.19 to 0.89)	-	0.41 (0.19 to 0.90)	-
Vomit	174 (20.8)	25 (11.2)	0.48 (0.25 to 0.89)	0.39 (0.17 to 0.90)	0.48 (0.25 to 0.90)	0.38 (0.17 to 0.84)
Dyspnea	63 (7.6)	168 (74.0)	34.8 (18.5 to 65.4)	32.64 (17.24 to 61.81)	34.84 (18.55 to 65.45)	32.53 (17.35 to 60.99)
Altered mental status	14 (1.7)	29 (12.8)	8.60 (4.62 to 16.01)	7.01 (1.94 to 25.27)	8.56 (4.60 to 15.95)	5.85 (1.44 to 23.85)
Seizures	29 (3.5)	17 (7.5)	2.25 (1.02 to 5.02)	-	2.24 (0.99 to 5.01)	-
Dehydration	39 (4.7)	28 (12.4)	2.86 (1.58 to 5.17)	2.60 (1.28 to 5.27)	2.83 (1.60 to 5.00)	2.55 (1.22 to 5.31)
Shock	5 (0.6)	23 (10.2)	18.72 (7.53 to 46.50)	-	18.63 (7.47 to 46.78)	-
